# Insights into metazoan evolution from *alvinella pompejana *cDNAs

**DOI:** 10.1186/1471-2164-11-634

**Published:** 2010-11-16

**Authors:** Nicolas Gagnière, Didier Jollivet, Isabelle Boutet, Yann Brélivet, Didier Busso, Corinne Da Silva, Françoise Gaill, Dominique Higuet, Stéphane Hourdez, Bernard Knoops, François Lallier, Emmanuelle Leize-Wagner, Jean Mary, Dino Moras, Emmanuel Perrodou, Jean-François Rees, Béatrice Segurens, Bruce Shillito, Arnaud Tanguy, Jean-Claude Thierry, Jean Weissenbach, Patrick Wincker, Franck Zal, Olivier Poch, Odile Lecompte

**Affiliations:** 1 Department of Structural Biology and Genomics, Institut de Génétique et de Biologie Moléculaire et Cellulaire (IGBMC), CERBM F-67400 Illkirch, France; INSERM, U596, F-67400 Illkirch, France; CNRS, UMR7104, F-67400 Illkirch, France; Faculté des Sciences de la Vie, Université de Strasbourg, F-67000 Strasbourg, France; 2 CNRS, UMR 7144, Adaptation et Diversité en Milieu Marin, Station Biologique de Roscoff, 29682, Roscoff, France; 3 UPMC Université Paris 6, Station Biologique de Roscoff, 29682, Roscoff, France; 4 Genoscope - Centre National de Séquençage, 2 rue Gaston Crémieux CP5706 91057 Evry cedex, France; 5 CNRS Institut Ecologie et Environnement (INEE), 3 rue Michel-Ange, 75794, Paris cedex 16, France; 6 UPMC Université Paris 6, UMR 7138, Systématique, Adaptation et Evolution, Campus de Jussieu, 75005 Paris, France; 7 Université Catholique de Louvain, Laboratoire de Biologie Cellulaire, Institut des Sciences de la vie, Croix du sud 5, B-1348, Louvain-la-neuve, Belgium; 8 UMR 7177 CNRS-UDS, LDSM2 Institut de Chimie de Strasbourg, 1 rue Blaise Pascal -BP 296 R8, 67008 Strasbourg cedex, France

## Abstract

**Background:**

*Alvinella pompejana *is a representative of Annelids, a key phylum for evo-devo studies that is still poorly studied at the sequence level. *A. pompejana *inhabits deep-sea hydrothermal vents and is currently known as one of the most thermotolerant Eukaryotes in marine environments, withstanding the largest known chemical and thermal ranges (from 5 to 105°C). This tube-dwelling worm forms dense colonies on the surface of hydrothermal chimneys and can withstand long periods of hypo/anoxia and long phases of exposure to hydrogen sulphides. *A. pompejana *specifically inhabits chimney walls of hydrothermal vents on the East Pacific Rise. To survive, *Alvinella *has developed numerous adaptations at the physiological and molecular levels, such as an increase in the thermostability of proteins and protein complexes. It represents an outstanding model organism for studying adaptation to harsh physicochemical conditions and for isolating stable macromolecules resistant to high temperatures.

**Results:**

We have constructed four full length enriched cDNA libraries to investigate the biology and evolution of this intriguing animal. Analysis of more than 75,000 high quality reads led to the identification of 15,858 transcripts and 9,221 putative protein sequences. Our annotation reveals a good coverage of most animal pathways and networks with a prevalence of transcripts involved in oxidative stress resistance, detoxification, anti-bacterial defence, and heat shock protection. *Alvinella *proteins seem to show a slow evolutionary rate and a higher similarity with proteins from Vertebrates compared to proteins from Arthropods or Nematodes. Their composition shows enrichment in positively charged amino acids that might contribute to their thermostability. The gene content of *Alvinella *reveals that an important pool of genes previously considered to be specific to Deuterostomes were in fact already present in the last common ancestor of the Bilaterian animals, but have been secondarily lost in model invertebrates. This pool is enriched in glycoproteins that play a key role in intercellular communication, hormonal regulation and immunity.

**Conclusions:**

Our study starts to unravel the gene content and sequence evolution of a deep-sea annelid, revealing key features in eukaryote adaptation to extreme environmental conditions and highlighting the proximity of Annelids and Vertebrates.

## Background

Annelids, commonly known as segmented worms, are typical triploblastic coelomate animals belonging to the Protostomes. Annelids, and especially polychaetous annelids, are important systems for understanding evolution and development in animals (for recent reviews, see [1,2]). Fossil records [3], as well as comparative morphology studies [4], suggested that the urbilaterian (the last common ancestor of bilateral symmetric animals) may have resembled annelids. Although such an assumption is difficult to verify, it is widely accepted that polychaetes exhibit many ancestral traits in their body plan and embryonic development [5].

Despite this long history as evo-devo model organisms, polychaete annelids, and more generally Lophotrochozoan representatives, are still poorly represented in sequence databases. Sequencing projects have mainly focused on Deuterostomes (Chordates and Echinoderms) and Ecdysozoa, i.e. molting Protostomes including arthropods and nematodes [6]. The recent enlargement and diversification of the sequencing project panel has played a decisive role in obtaining a more realistic picture of animal evolution. For instance, the analysis of genomic loci in the marine polychaete *Platynereis dumerilii *has revealed the intron-rich nature of annelid genes [7]. More recently, the genome of a bilaterian sister group, the cnidarian sea anemone *Nematostella vectensis*, has proved to be more complex than expected, with a gene repertoire, exon-intron structure, and large-scale gene linkage more similar to vertebrates than to flies or nematodes [8].

Among polychaete annelids, *Alvinella pompejana *[9], the "Pompeii worm", has attracted attention since it is currently considered to be one of the most thermotolerant eukaryotes on Earth, withstanding the largest known chemical and thermal ranges (from 5 to 105°C) [10-12]. This tube-dwelling worm forms dense colonies on the surface of hydrothermal chimneys and can withstand long periods of hypo/anoxia and long phases of exposure to hydrogen sulphides [13]. *A. pompejana *specifically inhabits chimney walls of hydrothermal vents on the East Pacific Rise [14,15]. It often co-occurs with *Alvinella caudata*, a very closely related species, and can be found in variable proportions according to the chemical conditions. The chimney walls are characterised by high flows of vent fluid, and therefore the highest temperatures for vent metazoans (temperatures usually range between 25 and 60°C, with exceptional bursts up to 105°C [10,11,13]), as well as high concentrations of potentially toxic compounds (e.g. H2S). The thermotolerance of alvinellid worms has been confirmed by laboratory observations of *Paralvinella sulfincola *thermotaxis [16]. To survive, *Alvinella *has developed numerous adaptations at the physiological and molecular levels, such as an increase in the thermostability of proteins and protein complexes [17-22]. As such, *A. pompejana *constitutes a precious source of thermostable proteins and macromolecular complexes of eukaryotic origin for the biochemical, biophysical or structural characterisation of proteins of fundamental or biomedical relevance. It has been selected as a model organism for structural studies by the Structural Proteomics IN Europe 2 (SPINE2) initiative. The pertinence of the model is confirmed by the recent study of the superstable superoxide dismutase recombinant protein [23]. The crystal structure at 0.99 Å resolution reveals anchoring interaction motifs in loops and termini, accounting for the enhanced stability of the *A. pompejana *protein compared to its human ortholog.

Here, we report the construction, sequencing and analysis of four full-length enriched cDNA libraries of *A. pompejana*. One of these libraries has been constructed from complete animals after removal of the dorsal tegument, which harbours an episymbiont community of Epsilonproteobacteria [24]. The other three libraries have been prepared from distinct tissues: the gills located at the anterior part of the animal and oriented towards the outside of the tube, the ventral tissues and the posterior region. This latter includes the pygidium and the subterminal growth zone of the animal where cell proliferation takes place. These three tissues, radically different with respect to their physiological roles, have been chosen in order to improve the transcriptome coverage. They also represent samples along the antero-posterior axis of *A. pompejana *since it has been reported that the body of the animal experiences a temperature gradient, the posterior part of *A. pompejana *being exposed to higher temperatures [10,12].

Analysis of the cDNA sequences allowed the determination of 9,221 putative protein sequences that were annotated using a new integrative functional annotation pipeline. The observed abundance of transcripts related to oxidative stress resistance, detoxification, anti-bacterial defence and heat shock protection, as well as the compositional bias detected in the protein sequences, may contribute to the adaptation of the Pompeii worm to its challenging habitat. From an evolutionary perspective, our analysis reveals striking similarities between Annelids and Deuterostomes, both in terms of sequence conservation and gene repertoires that raise interesting issues concerning the nature of the Bilaterian ancestor.

## Results and discussion

### cDNA libraries, EST assembly and cDNA characterization

Four non normalized libraries enriched in full length cDNA were constructed, with an average insert size of 2.5-3 Kb. The first library was prepared using whole adult individuals while the others were constructed from dissected tissues: gills, ventral tissue, and pygidium. A total of 100,177 chromatograms were processed by our semi-automated assembly pipeline (see Methods). 76% of the initial raw sequences fulfil our stringent quality criteria, and have a mean length of 674 bp (Table 1). The percentage of sequences exhibiting a poly(A) tail ranges between 8 and 12% in the different libraries, except in the ventral tissue library in which 42% of the sequences contain a poly(A) tail. The sequences were submitted to the EST section of the EMBL database under accession numbers FP489021 to FP539727 and FP539730 to FP565142.

**Table 1:** Summary of *A. pompejan a *cDNA libraries and assemblies

	CloneMiner		Oligo-capping		Global assembly
		
	Whole animal	Pygidium	Ventral tissue	Gills	
**Initial chromatograms**	20,549	36,648	16,411	26,569	100,177
**Clean sequences**	19,739 (96%)	25,419 (69%)	12,871 (78%)	18,105 (68%)	76,134 (76%)
3' poly(A) (%)	1,599 (8%)	3,156 (12%)	5,465 (43%)	1,467 (8%)	11,687 (15%)
Mean length (bp)	633	610	720	776	674
**Unigenes after assembly**	5,425	8,682	2,831	3,760	15,858
Contigs	1,365	2,327	917	1,193	4,993
Singletons	4,060	6,355	1,914	2,567	10,865
Contig mean length (bp)	993	951	852	931	1,017

The assembly of the 76,134 selected sequences (12.4 Mb) was performed by CAP3 using stringent parameters to avoid misassembly problems such as creation of chimeric contigs or combination of paralogs. We performed a global assembly of all sequences as well as a separate assembly for each library (Table 1). The global assembly yielded 4,993 contigs and 10,865 singlets (15,858 unigene sequences). Contig size ranges from 2 to 7,845 reads with a mean of 13 and a median of 3 (see Additional file 1, Figure S1). The average length of contigs is 1,017 bp. Taking into account our conservative approach, each contig and singlet of this assembly ideally represents a unique version of an expressed gene, i.e. paralogs, divergent alleles or splicing variants should not coalesce into the same contig.

The 15,858 unique sequences from the global assembly were analysed using two independent methods (ESTScan and a similarity-based approach) to determine the boundaries of the CoDing Sequences (CDS) and UnTranslated Regions (UTR). A total of 9,221 coding regions was obtained, including 2,932 complete CDS. The complete CDS lengths range from 90 nt to 2,694 nt with a mean of 540 nt (see the distribution of the corresponding protein lengths in Additional file 2, Figure S2). The mean GC content in the CDS is 46.2%. As previously observed in eukaryotic mRNAs [25-27], the mean GC content in *A. pompejana *is higher for the 5'UTR (45.7%) than for the 3'UTR (39.7% without poly(A) tail) and is comparable to the value previously observed in Annelids (43.7% and 34.1% respectively, data compiled from UTRdb [28]).

### *A. pompejana *genes undergo a neutral evolutionary process?

To investigate the evolutionary model driving the GC content in *A. pompejana*, an in-depth GC analysis was performed on the 84 almost complete mRNA coding for ribosomal proteins, including 15 genes expressed at low levels associated with the mitochondrial ribosome, and a set of genes with mid-to-high expression associated with the nuclear ribosome. The analysis shows that the GC3 content of CDS (0.481 ± 0.075) was significantly higher than the overall GC content of CDS (0.422 ± 0.029) and both UTR regions (0.389 ± 0.051; pairwise t-test, p < 0.0001). Unexpectedly, the GC3 was found to be constant, regardless of the length of the coding regions (F = 0.92, p-value = 0.341), even though a significant positive relationship exists between the cDNA length and its level of transcription (F = 18.18, p-value = 5 10-5) when estimated from the number of gene repeats in cDNA libraries. This number ranges from one (mt S proteins) to 223 copies (P0). Another striking result was the non linear evolution of the GC3 content of ribosomal protein transcripts with the level of gene expression. Both GC3 (CDS) and GC (UTRs) contents rapidly increased with the number of copies until they reached a plateau at a threshold value of c.a. 25-30 copies (see Additional file 3, Figure S3). The GC3 and GC content asymptotic values of CDS and UTR were close to 0.55 and 0.40, respectively. The correlation was significant (coding regions: F = 9.41, p-value = 0.003), although it was not significant when the mt ribosomal-protein genes were removed from the dataset. Analysis of codon usage in the ribosomal set revealed that eight of the most frequent codons are terminated by C or G (Phe, Leu, Tyr, His, Gln, Asn, Lys and Glu), seven by A or T (Ser, Pro, Thr, Asp, Cys, Arg, Gly) and three by two equally-frequent codons (Ile, Val and Ala). The search of optimal (Fop) codons using the factorial multivariate analysis tool in the CodonW software indicated that 7 AT-ended codons (TTA, CTA, ATA, TTT, AGT, GAA and TCA) were clearly associated with low-expressed mitochondrial ribosomal genes (first axis, inertia = 9.95% of the whole variance: data not shown). Five GC-ended codons (TAC, GCG, ATC, AAG, GCC) and CGT (Arg) were positioned at the other extremity of the first axis but without a clear relationship with the level of expression.

In general, synonymous codon usage biases are explained by two alternative but non exclusive models: a neutral mutational-bias and a selective model [29]. The expectation of the mutational-bias model corresponds to a positive relationship between the base composition of synonymous sites and their neighbouring silent sites (i.e. UTR and/or introns). In agreement with this model, we found a positive correlation between the GC3 and the GC(UTR), suggesting that both GC classes are evolving in the same way. The selective model postulates a co-evolution between synonymous codon usage and the abundance of tRNA to optimize the translation efficiency (notion of 'optimal' codons). According to Eyre-Walker [30], selection maximizes the speed of the translation and minimizes the costs of proofreading, resulting in a codon usage correlated with the expression level and the mRNA length. Such correlations have been observed in *Drosophila *and *Caenorhabditis *[31] but not in Vertebrates [29]. In *A. pompejana*, there is no correlation between the GC3 and the level of gene expression within the set of nuclear ribosomal genes used. Even if differences were observed when considering the level of ribosomal gene expression, this was mainly due to the presence of two distinct sets of ribosomal protein genes (i.e. mitochondrial ribosome versus nuclear ribosome). Such a result also held for the Fop codons since no clear gradient of expression was found among the nuclear ribosomal genes along the first axis of the COA although the preferred AT-ended codons were clearly associated with the mitochondrial ribosomal genes. Additionally, no correlation was found between GC3 and cDNA length. Thus, there is no evidence for a selective process acting on silent sites although extended analyses on GC content bias over the genome and the whole transcriptome [32] are clearly necessary to validate the predominance of the neutral model in *A. pompejana*.

### Integrative functional annotation

The 7,353 protein sequences from the global assembly that exhibit homology in the protein databases were analysed using a new integrative functional annotation pipeline (Gagnière et al., manuscript in preparation). The originality of this pipeline lies in the exploitation of the evolutionary context of the protein sequences based on a clustered Multiple Alignment of Complete Sequences (MACS) [33]. The sequences are thus analysed in the framework of the overall family and subfamily, allowing a reliable propagation of sequence annotations in conserved regions. Thanks to this novel pipeline, 6,252 (85.0%) of the 7,353 protein sequences with homology were annotated with either a text mining definition, an EC number, Pfam-A domains or a Gene Ontology term (Table 2).

**Table 2:** Overview of the annotation results.

Level 1 annotations	Number of proteins (%)
Proteins with homologs	7,353 (100%)
Pfam-A domains	4,767 (65%)
Gene Ontology	5,949 (81%)
GO Biological Process	5,072 (69%)
GO Cellular Component	4,530 (62%)
GO Molecular function	5,601 (76%)
Text mining definition	4,611 (63%)
Enzyme classification	1,243 (17%)
EC level 4 (X.X.X.X)	1,180 (16%)
Annotated proteins	6,252 (85%)

**Level 2 annotations**	**Number of networks**

KEGG pathways	345
Mapped pathways	202
Coverage > 50%	82
STRING subnetworks	385
Mapped subnetworks	264
Coverage > 50%	63

This primary annotation was used to map the *A. pompejana *query proteins to the networks of the KEGG and EMBL STRING databases. The reconstructed metabolic pathways and interaction networks constitute an integrative second level annotation which is essential to the study of the biological processes at work in *A. pompejana*. Among the 345 metabolic reference pathways of the KEGG database, 82 pathways (40.6%) have been populated by *A. pompejana *proteins at a level greater than 50% (in terms of number of distinct enzymes). Since the reference pathways are highly redundant (several enzymes can catalyse the same reaction), the *A. pompejana *cDNA appear to provide a good coverage of a large panel of metabolic pathways. Common pathways such as glycolysis, gluconeogenesis, citrate cycle, purine and pyrimidine metabolism are functionally complete or almost complete. More specific pathways are also well represented, such as androgen and estrogen metabolism or steroid biosynthesis, as illustrated in Additional file 4, Figure S4. 3,582 *A. pompejana *proteins (48.1%) have also been mapped to human networks in the STRING database, which were first cut into smaller sub-networks (see methods). 68.8% of these human sub-networks were populated by at least one *A. pompejana *ortholog.

In the absence of a complete annotated genome of a close relative of *A. pompejana *and considering the diversity of genome size reported in polychaete annelids (from 58 Mb to 7 Gb [34], *A. pompejana *being intermediate with 675 Mb [35] for 32 chromosome pairs [36]), we cannot estimate the coverage of our cDNA libraries. However, the mapping of *A. pompejana *proteins on KEGG metabolic pathways and on STRING networks emphasizes the broad coverage of our cDNA databases and provides an integrative framework for the interpretation of *A. pompejana*'s proteome features.

### *A. pompejana *database and website

The sequences and annotation features are stored in a relational database [37] that maintains fine grained information about (i) the library origin of each clone, (ii) the nucleic sequences and their phred quality values, (iii) the different assemblies and their associated parameters, (iv) the predicted protein sequences and (v) all the results of the annotation process.

**Figure 1:**
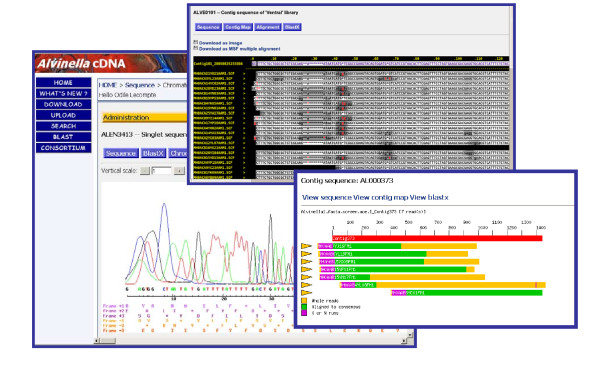
**Screenshots of the Alvinella website illustrating some of the visualisation tools**.

The data are accessible via a user-friendly web interface. To allow intuitive querying of the database and convivial data visualisation, we developed dedicated tools, ranging from trace visualization to the interactive display of the alignment of *A. pompejana *protein sequences and their families (Figure 1). Two modules are available for querying the database: a homology search module using NCBI BLAST and a module for performing full-text searches of all annotations. Subsets of relevant data can then be selected from the search results for display purposes. Different views are available: (1) nucleic cDNA sequence, (2) EST trace and six-frame translation, (3) contig schematic representation, (4) Consed-like [38] contig alignment view, (5) MACSIMS annotated protein alignment with customisable features display, (6) integrative view of the annotation process (text mining definition, EC number, Gene Ontology, Pfam-A domains, KEGG pathway mapping, EMBL STRING mapping).

### Adaptation to hypoxia, oxidative stress and heavy metals

**Figure 2:**
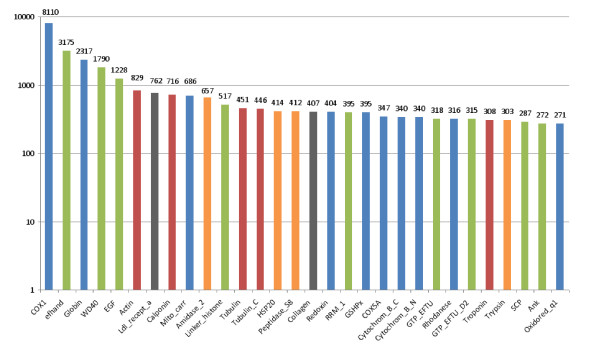
**The 30 most frequent PFAM-A domains identified in *A. pompejana *protein sequences**. The Y-axis indicates the total number of reads encoding proteins with this domain. We used a logarithmic scale for representation constraints. Bars are coloured according to functional role categories: respiration, oxidative stress and/or detoxification in blue, protein-protein or protein-DNA/RNA interactions in green, structural proteins in red, anti-bacterial defence in orange and others in grey.

As an endemic species of the hydrothermal vent ecosystem colonizing the chimney walls, *A. pompejana *has to deal with very variable conditions that are the result of a chaotic mixing of vent fluid (350°C, anoxic, CO2- and sulphide-rich) and deep-sea water (2°C, mildly hypoxic). In this environment, oxygen and CO2 concentrations, pH and sulphide levels vary quickly and over a wide range. These challenging environmental conditions seem to be reflected in the highly expressed gene pool detected using two complementary indicators: the size of the cDNA clusters (in terms of the number of reads) (Table 3) and the abundance of transcripts corresponding to a PFAM-A domain (Figure 2). Domains involved in protein-protein or protein-DNA/RNA interactions are particularly abundant (calcium-binding domain EF-hand, WD-40 repeat, RNA recognition motif RRM_1, ankyrin...), as frequently observed in studies of eukaryotic transcriptomes [39-41]. The highly expressed genes also include genes encoding extracellular structural proteins, such as collagen, as well as cytoskeleton proteins (actin, myosin, tropomyosin, calponin, tubulin, troponin). More interestingly, most abundant transcripts encompass genes clearly linked to oxygen homeostasis, oxidative stress resistance, detoxification and to a lesser extent, antibacterial defense and heat shock protection. Considering the stress of depressurization, dramatic temperature decrease, and the general trauma endured by animals during sampling, the transcript abundance should be interpreted cautiously. A more detailed analysis using species-specific oligo microarrays on a set of specimens from an isobaric collection would be required to safely deduce the precise role of a given gene in the environmental response of the worm. Nevertheless, highly expressed genes related to environment provide interesting clues about general aspects of the worm adaptation.

**Table 3:** Highly expressed genes in *A. pompejana *libraries

Access	Fonction	Reads*
TERA04282	Cytochrome c oxidase subunit 1 (EC 1.9.3.1)	7845
TERA02741	Hypothetical protein	2029
TERA02189	Hypothetical protein	917
TERA02142	Hypothetical protein	879
TERA03177	Actin	533
TERA00344	Extracellular globin (Haemoglobin A2 chain precursor)	524
TERA02067	Hypothetical protein	424
TERA00650	Hypothetical protein	422
TERA03305	Intracellular haemoglobin	386
TERA00833	Extracellular haemoglobin (Haemoglobin B2 chain precursor)	370
TERA00205	Extracellular haemoglobin linker L1	349
TERA03100	Cytochrome c oxidase subunit 5A (EC 1.9.3.1)	344
TERA00354	Cytochrome b	338
TERA04769	Hypothetical protein	335
TERA00845	Extracellular haemoglobin (Haemoglobin B1 chain precursor)	322
TERA02090	Hypothetical protein	304
TERA01907	Small heat shock protein (sHSP)	295
TERA02261	Myosin essential light chain	285
TERA01929	Hypothetical protein	275
TERA00421	Extracellular haemoglobin linker L3	267
TERA03231	Glutathione peroxidase	264
TERA01189	Hypothetical protein	263
TERA02903	Hypothetical protein	258
TERA01828	Tropomyosin	232
TERA00984	Hypothetical protein	230
TERA02160	Elongation factor 1-alpha	218
TERA01465	Peptidoglycan recognition protein	200

*The last column indicates the number of reads in the global assembly.

Among these genes, the most important fraction corresponds to proteins from the respiratory chain and three main types of hemoglobins (Hb) reported in Alvinellidae, namely a non-circulating cytoplasmic globin, the extracellular giant annelid hexagonal bilayer HBL-Hb of the vascular system and the circulating intracellular Hb found in the coelomic fluid (for a review, see [42]). The abundance of Hbs in *A. pompejana *and their high oxygen affinities [43] may be determinant in the respiratory adaptation to hypoxic/anoxic environments. Interestingly, the set of highly expressed genes includes several "hypothetical proteins" (Table 3). They exhibit sequence segments with a biased residue composition and have no significant similarity with known proteins, except in some cases for low complexity regions. One of these specific proteins, TERA02189, belongs to a family of proteins that is highly conserved in *A. pompejana*. A comparative proteomics study [44] revealed that three members (TERA02082, TERA02935 and TERA08242) of this family are differentially expressed depending on the oxygen concentration. These oxygen-responsive genes represent potential candidates that may contribute to oxygen homeostasis.

Despite the hypoxia encountered in its environment, the Pompeii worm can be subject to exogenous oxidative stress [45]. High levels of ferrous iron and sulphide have been reported to favour the formation of reactive sulphide species (RSS), an analog to ROS. This is in agreement with the high level of expression observed for the major antioxidative enzymes in *A. pompejana*: Mn and Cu/Zn superoxide dismutases, peroxiredoxins, glutathion peroxidases (GPX), thioredoxin. Interestingly, no catalase cDNA was detected in our set. Although we cannot exclude the existence of a catalase gene (possible low expression of this gene), the absence of cDNA in our sample suggests that H2O2 formation might not be the most common mechanism of detoxification. An earlier study [45] also questioned the presence of catalase in *Paralvinella grasslei*, a close relative of *A. pompejana*. The authors suggested that alternative H2O2-scavengers, such as antioxidant osmolytes or other enzymes might replace the catalase activity. Indeed, taking into account the diversity and level of expression of glutathione peroxidases and peroxiredoxins in *A. pompejana*, SOD-derived H2O2 could be degraded by peroxidases rather than by catalases as suggested by Dixon and colleagues [46].

In addition to hypoxia and oxidative stress, *A. pompejana *has to deal with large amounts of heavy metals. Invertebrates possess a variety of cellular detoxification pathways that reduce the concentrations of potentially toxic metals circulating in the blood (reviewed in [47]). These pathways include metal binding by cysteine-rich proteins known as metallothioneins, followed by their elimination through the lysosomal endomembrane system. We detected a single EST coding for a metallothionein-like protein in our library suggesting that involvement of metallothioneins is not the major detoxication process. However, we cannot exclude the possibility that the pool of highly expressed genes of unknown function contains genes coding for new metallothionein-like proteins, since the pool of unknown genes appears to be enriched in cysteine-rich proteins. Another alternative for heavy metal detoxication is the intracellular sequestration in specific vacuoles producing solid granules [47]. This would be in agreement with the presence of arsenic, zinc and copper detected in *A. pompejana *epidermal cells [48] and the production of a large amount of iron-containing granules by *A. pompejana *mucocytes [49]. Finally, rhodanese also appears to be preponderant among the highly expressed genes. Rhodanese can perform a variety of roles (reviewed in [50]), including the modulation of general detoxification processes and the maintenance of redox homeostasis.

### Thermo-adaptive features in amino-acid composition

As one of the most thermotolerant eukaryotes known to date, the Pompeii worm clearly provides a unique model for the study of adaptation to high temperature in this domain of life. Its thermal regime generally fluctuates between 25 and 60°C, with exceptional bursts up to 105°C [11,13]. These high and variable temperatures require adaptations at the physiological and molecular levels, even though we are far from the optimal temperature range reported in hyperthermophilic prokaryotes. At the molecular level, several studies have revealed the higher thermostability of *A. pompejana *proteins and complexes compared to their orthologs from other eukaryotes [17-23]. We thus investigated the composition of *A. pompejana *proteins and found a biased composition compared to their orthologs from 5 major Metazoa lineages (Vertebrates, Arthropods, Nematodes, Platyhelminthes, Cnidaria) (Additional file 5, Figure S5). The amino acid composition differed significantly among taxa (Homogeneity statistic: χ2 = 0.01153 G = 0.01153). *A. pompejana *exhibits the highest proportion of charged amino acids (nearly 25.5%): a characteristic also shared by the cnidarian *N. vectensis *(25.4%). This is mainly due to an increase of the positively-charged amino acids lysine and arginine (12.6%). This excess of charged residues may enhance protein stability in thermophilic eukaryotes, notably by increasing salt-bridges, if they can interact with negatively-charged residues. This would be in keeping with the structural analysis of the superoxide dismutase of *A. pompejana *[23], suggesting that extra salt-bridged interactions may be involved in the superstability of this protein.

In prokaryotes, thermoadaptive molecular features appear to be multiple and variable [51-56]. Berezovsky and Shakhnovich [55] suggested two distinct evolutionary strategies to conciliate these conflicting observations. In prokaryotes with an ancestral thermophilic character (e.g. Archaea such as *Pyrococcus*), proteins may be significantly more compact and more hydrophobic than their mesophilic counterparts. Conversely, organisms that recently colonized a hot environment such as the bacteria *Thermotoga maritima*, may have evolved under a more ''sequence-based'' mechanism of thermostability. In this latter case, a few charged amino acid replacements or amino acid deletions increased occurrences of hydrogen bonds and inter-subunit electrostatic interactions or decreased the length of surface loops respectively. The enrichment in charged residues detected in *A. pompejana *suggests that the sequence-based mechanism of thermostability reported to hold in *Thermotoga *may also apply to *A. pompejana*. However, the molecular basis of eukaryotic thermotolerance is probably more complex than that of the bacterial/archaeal domains, and additional studies are needed to decipher the molecular basis of thermostability in *A. pompejana*. These may include massive structural comparisons between *A. pompejana *proteins and mesophilic orthologs, as well as in-depth comparisons of amino acid compositions between close relatives of *A. pompejana *since thermoadaptive features are often masked by a background of evolutionary sequence divergence.

### Proximity between Annelids and Vertebrates

**Figure 3:**
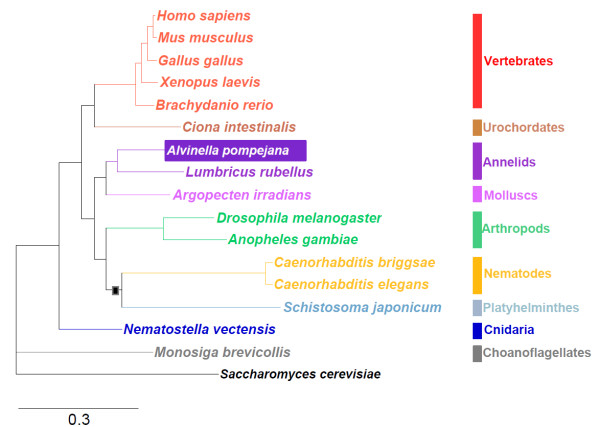
**Bayesian phylogeny of Metazoa**. The analysis was performed on a concatenation of 76 ribosomal protein family alignments, with *Saccharomyces cerevisiae *as the outgroup. The scale bar indicates the expected number of amino acid substitutions per aligned position. All nodes were resolved in 100% of the sampled topologies from the Bayesian analysis, except the node indicated by a square (support value of 96%).

Contradictory results have been obtained in whole-genome based phylogenetic studies that favour either the classical "Coelomata hypothesis" positioning Nematodes and Plathyhelminthes as early branching clades [57,58] or the new animal phylogeny dividing Protostomes in Ecdysozoa (including Arthropods and Nematodes) and Lophotrochozoa (including Annelids, Molluscs and Platyhelminthes) [8]. To address the evolutionary relationships between *A. pompejana *and other animals, we first investigated the phylogenetic position of *A. pompejana *using a pool of 76 ribosomal proteins. A phylogenetic tree reconstruction was performed on the concatenation of the corresponding multiple alignments using MrBayes (Figure 3). The obtained topology, together with a maximum parsimony analysis (data not shown), supports the phylogenetic tree proposed by Aguinaldo *et al.*, with the exception of *Schistosoma japonicum *(Platyhelminthes) that clustered within the Ecdysozoa represented by Arthropods and Nematodes, rather than Lophotrochozoa (represented by Molluscs and Annelids).

Compared to other Protostomes used in this study, the annelids *A. pompejana *and *Lumbricus rubellus *and the mollusc *Argopecten irradians *show relatively small branch lengths. Thus, the slow evolutionary rate previously observed in diverse species of the polychaete lineage [6,7] seems also to hold in *A. pompejana*, despite its challenging habitat. In contrast, fast evolutionary rates are observed in the parasitic worms *Schistosoma *or *Caenorhabditis*, that might lead to a long-branch attraction artefact between these two lineages in the present analysis and partly explain the contradictory results obtained in whole-genome based phylogenetic studies. Differences in the rate of evolution are reflected in the mean percent identity between orthologs observed in a pool of 556 unambiguous ortholog families (86,727 positions without gaps) conserved in 6 major Metazoa lineages (Table 4). *A. pompejana, Homo sapiens *and *N. vectensis *exhibit high sequence conservation relative to *S. japonicum *or *C. elegans *while *D. melanogaster *appears intermediate. Thus, the proximity between annelid and Vertebrate sequences previously reported for proteins from the polychaete *Platynereis dumerilii *[7] is now observable at a larger scale and can be partly extended to Cnidaria.

**Table 4:** Mean percent identities between orthologous protein sequences of Metazoa.

	Ap	Hs	Dm	Ce	Sj	Nv
*Alvinella pompejana *(Ap)	100,0					
*Homo sapiens *(Hs)	65,9	100,0				
*Drosophila melanogaster *(Dm)	62,7	61,7	100,0			
*Caenorhabditis elegans *(Ce)	56,3	55,3	55,1	100,0		
*Schistosoma japonicum *(Sj)	57,7	56,0	54,8	51,0	100,0	
*Nematostella vectensis *(Nv)	65,1	64,5	60,8	54,9	55,3	100,0

### Differential gene losses

The proximity between *Alvinella*, Vertebrates and Cnidaria discussed above is also observable in the gene repertoire of the Pompeii worm. Only 135 protein families present in *A. pompejana *are specific to Protostomes. Among them, only 13 are conserved in all the Arthropod, Nematode and Platyhelminthe representative species, illustrating the prevalence of differential gene losses in Protostomes. Moreover, only 5 proteins in our dataset are specific to Annelids and Platyhelminthes. As we only have access to the proteome of a parasitic Platyhelminthe, this apparent split may actually be the consequence of massive gene losses in *Schistosoma *linked to its parasitic lifestyle. This illustrates the urgent need for the sequencing of complete genomes from free-living species of Platyhelminthes, and more generally from diverse representatives of Lophotrochozoa, in order to identify the relationships and synapomorphies unifying the Spiralia (Annelids and Molluscs) and Platyhelminthes within Lophotrochozoa. Considering the high proportion of *A. pompejana *"specific" genes (20%), these genomes would be especially valuable for the discrimination of genes that are truly specific to Alvinellidae (and possibly linked to environmental adaptation) and those that are in fact shared by other lineages of Lophotrochozoa.

In contrast to the small sets of proteins specific to Protostomes, 203 *A. pompejana *proteins belong to families or superfamilies specific to Deuterostomes. This Deuterostomia-set is significantly enriched in glycoproteins (34 proteins out of 203) that play a key role in many biological processes, in particular intercellular communication and adhesiveness, hormonal regulation, or immunity. For instance, the protein TERA08399 belongs to the superfamily of secreted cysteine rich factors and its N-terminal domain sequence exhibits the idiosyncratic features of the IGFBP (Insulin-Like Growth Factor Binding Protein) family reported to be vertebrate-specific [59]. Another noteworthy result is the enrichment in proteins containing an epidermal growth factor (EGF)-like domain that is frequently found in the extracellular part of membrane-bound proteins or in proteins known to be secreted. In addition, the Deuterostomia-set is enriched in proteins involved in the I-kappaB kinase/NF-kappaB cascade or in death-domain containing proteins that can be involved in the regulation of apoptosis and inflammation or linked to innate immunity. This includes close homologs of the CRADD and DEDD/DEDD2 protein families (TERA03000 and TERA04373, respectively) that play a role in the stress-induced apoptosis signalling pathway and are important mediators for death receptors [60,61]. If we exclude the possibility of horizontal gene transfer, these genes encoding important functions previously considered as specific novelties of Deuterostomes were in fact already present in the Bilaterian ancestor and were subsequently lost in Ecdysozoa and Platyhelminthe model species or diverged beyond recognition in the representative species of Ecdysozoa and Platyhelminthes used in our study.

In addition to this Deuterostomia-set, 147 *A. pompejana *protein families are specifically present in both Deuterostomes and Cnidaria, while 32 are specifically found in both Cnidaria and at least one Protostome. These 147 families present in the last common ancestor of the Eumetazoa may also have been lost in the Ecdysozoa and Platyhelminthe representatives. Interestingly, this set exhibits an enrichment in the selenium binding function. Notably, it includes the ortholog of the selenoprotein N involved in the regulation of oxidative stress and calcium homeostasis [62]. Differential gene losses in Ecdysozoa are also observed for more ancestral genes. For instance, the Pompeii worm possesses orthologs of the component of the phagocytic NADPH oxidase (Nox) (gp91phox and p22phox) and of some of its regulatory proteins (p47phox, p67phox) that play a critical role in innate immunity of Deuterostomes. p47phox and p22phox genes are present in the Cnidaria *N. vectensis *and the unicellular choanoflagellate *M. brevicollis*, but are absent in several lineages of ecdysozoans including *Drosophila *and *Caenorhabditis*.

Indeed, with the multiplication of genome and EST sequencing projects in invertebrates, many "vertebrate novelties" have been shown to be present in Cnidaria and/or Placozoa, but lost in the canonical model Protostomes, i.e. *D. melanogaster *and *C. elegans *[8,63-66]. There is now an increasing body of evidence supporting the prominent role of lineage-specific losses in animal evolution (for a review, see [1]), especially in Protostomes. The present analysis suggests that massive losses are not a shared trait of the Protostomes, since genes involved in major metazoan functions are retained in *A. pompejana*, a Lophotrochozoan representative. However, we cannot exclude that losses exist in Annelids and/or Molluscs. For instance, no enzyme involved in urea excretion has been identified in the *Alvinella *database, as expected from previous studies reporting an incomplete or non-functional urea cycle in a number of annelid species (loss of the citrulline-arginine segment, see [67]).

Genes are not the only genome features differentially lost in the course of Metazoan evolution, as suggested by a study of genomic regions of the Polychaete *Platynereis dumerilii *that revealed intron-rich genes in Annelids [7]. According to the authors' estimates, two-thirds of human introns would have been present in the bilaterian ancestor and retained in Annelids, while lost in the insect and Nematode genomes. The hypothesis of an intron-rich Bilaterian ancestor (discussed in [68]) has been extended to the ancestor of Metazoa through the examination of the exon-intron structure of *Nematostella *and *Trichoplax *genes [8,66]. Thus, the emerging picture of evolution is one of a complex ancestor of Metazoa, with a gene toolkit and a gene structure closer to those of extant Vertebrates and Annelids than to model Ecdysozoa. This contradicts the intuitive view of a linear evolution, from simple ancestral networks to more complex ones in Vertebrates, although it is in line with several studies suggesting a reductive evolution from a complex community of ancestors as a general trend in the evolution of life (see [69] and references therein).

## Conclusions

The construction and sequencing of four non normalized cDNA libraries from *A. pompejana*, one of the most thermotolerant eukaryotes known to date, resulted in 15,858 unique cDNA sequences and 9,221 annotated protein sequences. As indicated by the pathways and interaction networks mapped, our cDNA libraries provided a good coverage of the *A. pompejana *gene repertoire. Our analysis revealed that, apart from house-keeping genes, most abundant annotated transcripts were directly related to adaptation to the challenging physico-chemical conditions encountered by *A. pompejana*, in particular hypoxia, oxidative stress and heavy metals. In addition to these annotated genes, we also detected an important pool of unknown, specific and highly expressed genes that represent valuable targets for the study of *A. pompejana *adaptation. We also detected a compositional bias that may enhance protein thermostability in this eukaryote facing an extreme and variable thermal regime.

From an evolutionary perspective, our analyses support the new animal phylogeny and seem to indicate a slow evolutionary rate in *A. pompejana *despite its challenging environmental conditions. Moreover, we found that an important pool of ancestral genes involved in major metazoan functions are lost in other representatives of Protostomes but retained in *A. pompejana*, suggesting that massive losses are not a shared trait of the Protostomes. Sequence conservation together with ancestral gene retention identified a surprising proximity between *A. pompejana *and Deuterostomes. This makes *A. pompejana *thermostable proteins outstanding models for the study of human protein targets.

Special attention has been paid to making the sequences, assembly and annotations accessible via a user-friendly web site. They represent a significant contribution to the successful exploitation of *A. pompejana *proteins in the future annotation of genomes from annelids and related phyla and will hopefully stimulate future research on metazoan evolution and adaptation.

## Methods

### cDNA libraries and EST sequencing

*A. pompejana *samples were collected during the Biospeedo 2004 oceanographic cruise on the south East Pacific Rise at latitudes ranging from 14°S to 21°33S (25 individuals of which 6 were dissected on board). Animals were collected with the telemanipulated arm of Nautile in insulated boxes (4-5°C), recovered at atmospheric pressure but dissected a few minutes after being recovered onboard in RNALater stabilization and storage solution. All individuals and/or tissues were conserved in liquid nitrogen. Four non-normalized and full-length-enriched cDNA libraries were constructed at the CNS Genoscope. One was prepared from 6 whole animals while the others were constructed from specific tissues: gills (5 individuals), ventral tissue (5 individuals) and pygidium (3 individuals). The whole animal and pygidium libraries were constructed with the CloneMiner cDNA construction kit (Invitrogen), which is designed to construct cDNA libraries without the use of traditional restriction enzyme cloning methods. This technology combines the action of SuperscriptII reverse Transcriptase with the Gateway Technology. Single-stranded mRNA was converted into double stranded cDNA containing attB sequences on each end. Through site-specific recombination, attB-flanked cDNA was cloned directly into attP-containing donor vector by homologue recombination. The gill and ventral tissues libraries were prepared using the oligo-capping approach. Full length RNAs were enriched by the action of the bacterial alkaline phosphatase to digest 5'-uncapped mRNAs. A 30-mer 5' oligo was linked using T4 RNA ligase after removing the 5'cap using Tobacco acid pyrophosphatase. The first strand cDNA was primed with an oligo(dT)-SfI primer and double stranded using specific 5' and 3' primers and amplified by PCR. The PCR SfI-digested cDNA products were size selected to exclude fragments smaller than 1 kb and then linked into pME18S-FL3 DraIII-digested vector. A total of 100,177 different clones were sequenced.

### EST filtering, assembly and clustering

We developed a semi-automatic pipeline (TCL scripts) to manage and process the data, from the chromatograms to the assembled sequences. The raw SCF chromatogram files were clipped with respect to quality, repeats, and vector content. Quality clipping was based on phred [70,71] quality values: a window of size 20 bp was slid through the quality files from both sides, and the clip positions (left/right) were determined by the first window position with a phred-value above a threshold of 13. Vector masking was performed by cross_match against the UniVec database [72] and the pME18S and pDONR222 vector sequences. Masked sequences were cleaned from empty vector sequences and short sequences (<100 nt) were filtered out. To avoid misassembly, 5' poly(A) and 3' poly(T) sequence boundaries were masked using a 20/25 nt sliding window (in-house TCL script).

The EST assembly was performed using cap3 [73] with default parameters, with the exception of 'overlap percent identity cutoff' (-p) and 'clipping range' (-y) parameters set to 90% and 30 nt, respectively. The assembly was performed independently on each library as well as on the full complement of sequences.

### cDNA characterization

Complete and partial CoDing Sequences (CDS) were determined from assembled sequences using two independent approaches, similarity and *ab initio *prediction. Contig and singleton sequences were compared to protein sequences of the UniprotKB [74] and PDB [75] databases using BLASTX [76]. Coding frames were deduced from BLASTX best hit alignments (E-value ≤ 1e-05). Then, the CDS were created by extending the matching region in both 5' and 3' directions to the end of the cDNA sequence or a stop codon. If a stop codon was encountered in the 5'end, the first ATG codon following this stop codon was chosen as the initiation codon. When a frameshift was detected in the cDNA sequence, the translation of the incriminated region was replaced by masking symbols.

In parallel, we used ESTScan [77] to detect CDS. Since no large set of coding and noncoding sequences of annelids or molluscs are available for training, we used the *H. sapiens *model. In order to optimise a threshold for the ESTScan score, we established the distribution of sequences with or without homology according to ESTScan cut-off values. By setting an optimal cut-off value ≥ 200, we obtained a specificity of 70% and a sensitivity of 66%. To be considered as complete, a CDS must start with an initiation codon and end with a stop codon. Additionally, for CDS sequences deduced from BLASTX, the protein sequence must cover at least 80% of the best BLASTX hit.

The GC content study on mRNA encoding ribosomal proteins was performed for 84 almost complete cDNA (including 15 mitochondrial cDNA). The GC content was plotted against the number of repeats and subsequently tested with several regression models (linear, exponential, logarithmic and power) using the software SigmaPlot. The model that best fitted the dataset was a power function (y = ax+b). A search of optimal (Fop) codons was performed using a multifactorial correspondence analysis (CoA) of codon usage implemented in the CodonW software [78,79].

### Integrative functional annotation

MACS [33] protein alignments were generated with the PipeAlign [80] toolkit. Integrative annotation was based on the MACSIMS [81] and GOAnno [82] software frameworks. MACSIMS divides the multiple alignments into subfamilies according to conservation patterns. It then validates or corrects functional and structural information mined from public databases before propagation to the query sequence. Pfam-A [83] annotations are extracted from MACSIMS. GOAnno provides Gene Ontology [84] annotations for the query, after analysis of the GO terms obtained for the query subfamily. In addition to these programs, we have developed new software (Gagniere et al., manuscript in preparation) to: (1) generate a text mining functional definition from close orthologs, (2) generate a consensus Enzyme Commission number from close orthologs, (3) map the annotated proteins to the KEGG (Kyoto Encyclopedia of Genes and Genomes) pathways [85] and the EMBL STRING (Search Tool for the Retrieval of Interacting Genes/Proteins) database [86].

The mapping of *A. pompejana *proteins to the STRING database was performed by retrieving data for the closest human Uniprot homolog. This homolog was then used to search the STRING database using different identifiers (Uniprot ID, Uniprot Ensembl, RefSeq and Genome Reviews accession numbers and gene names in this order). If no STRING homolog was found using this textual search, a BLASTP search was performed on STRING human protein sequences and the first best hit (E-value ≤ 1e-05) was chosen. Then, STRING networks were built (combined score cut-off ≥ 0.9) and sub-networks were extracted by retrieving level 1 neighbours for each protein. These small sub-networks were scored by the Ratio of consistency (Rc), defined as the ratio between the observed number of sub-network edges (protein-protein interactions) and the maximum theoretical number of edges. Rc will be high in sub-networks exhibiting a high level of intra sub-network interactions. In contrast, a low ratio indicates large sub-networks with few intra sub-network interactions. In order to reduce the set of sub-networks, two sub-networks A and B were fused if they matched the following criteria:

$$\begin{array}{c} 1-\left(1-\text{Nc}\right)\times \left(1-\text{RcA}\right)\times \left(1-\text{RcB}\right)\geq 0.7\\ \text{Nc}\geq 0.5 \end{array}$$

with Nc the ratio between the number of A and B shared nodes and the number of nodes in the smaller sub-network. These criteria help to preferentially fuse highly related sub-networks, while avoiding low consistency sub-networks that would otherwise agglomerate weakly related sub-networks. The final sub-networks were visualized using Cytoscape [87].

### Functional enrichment

The functional annotation clustering tool in the DAVID [88] software was used to study the the sets of differentially conserved genes. For each set, the closest homologs of the *A. pompejana *proteins from a given species (depending on the set under study) were processed against the background of this species. Enrichment with a P-value ≤ 0.01 was considered to be significant.

### Phylogeny and molecular evolution

Phylogenetic reconstruction was performed on 17 model taxa covering the main eukaryotic lineages (choanoflagellate: 1, cnidaria: 1, platyhelminthe: 1, nematod: 2, arthropod: 2, lophotrochozoa: 3 including *A. pompejana*, chordate: 6). The tree was rooted with the yeast *Saccharomyces cerevisiae*. The phylogenetic tree and rates of amino acid substitution for each branch were inferred on a concatenated alignment of 76 ribosomal protein families using MrBayes 3 [89] under the WAG model.

Both observed and simulated amino-acid frequencies associated with the orthologous set of protein coding regions (65167 amino acids) were obtained using the codeML package of the software PaML v3.14 [90] and the 'universal' genetic code. Amino-acid alignments were validated manually, concatenated and exported in a PHYLIP format using the software Se-AL v2.0 [91]. Regions containing gaps, misalignments or uncertainties were excluded from the analysis. PAML analyses were performed using a reference tree previously obtained from the ProML package of PHYLIP 3.68 [92] for the 9 taxa, using the JTT model of amino-acid substitutions. Amino acid frequencies were calculated using the aaML package (aadist = 'equal', with the jones.dat matrix) and standard deviations of frequencies were obtained from 100 rearrangements (bootstrap) of the dataset. This allowed us to estimate the proportion of hydrophobic, positively-charged and negatively-charged amino acids associated with the translated sequences across taxa, and to calculate hydrophobicities using the hydrophobic index based on the OMH scale of Sweet & Eisenberg [93]. This index is known to take into account the ability of an amino acid to be replaced by another during the course of evolution.

### Comparative genomics

Sequences were compared to the Uniprot database using BlastP and classified according to the taxonomy of their hits. Taxonomic groups taken into account include at least one species with a complete proteome represented in Uniprot: Deuterostomia, Nematoda, Arthropoda, Platyhelminthes, Cnidaria, other Metazoa, Choanoflagellida, "protists" (Alveolata, Diplomonadida, Cryptophyta, Entamoebidae, Euglenozoa, Mycetozoa, Parabasalidea, Rhodophyta and Stramenopiles), Viridiplantae, Fungi, other Eukaryota, Prokaryotes and Viruses. Hits obtained in Annelids or Mollusca were ignored. When all BlastP hits (E-value ≤ 1e-05)) of an *A. pompejana *protein were restricted to a unique taxon (with the exception of Annelids and Molluscs), this protein was considered to be specific to this taxon and Annelids (and potentially Molluscs).

### *Alvinella *database and website

The database is managed by the PostgreSQL relational database management system and is backed-up on a weekly basis. The Alvinella website uses an Apache HTTP server and PHP5 and was built from scratch using the Smarty template engine, the ADOdb database abstraction library and the phpGACL generic access control list library.

### Database accession numbers

The sequences have been submitted to the EST section of the EMBL database under accession numbers FP489021 to FP539727 and FP539730 to FP565142.

## Authors' contributions

DJ, FG, DH, BK, FL, ELW, DM, JFR, BS, JCT, FZ, OP and OL were founding members of the project. FZ provided biological samples. BS, CDS, JW and PW were involved in the construction and sequencing of cDNA libraries. NG, YB, DB, EP replicated the cDNA libraries. NG, OP and OL carried out the sequence assembly and annotation, the database and website construction, the gene expression studies and the phylogenetic and comparative genomics analyses. DJ carried out the GC and codon analyses. DJ and NG performed the search for thermo-adaptive features in amino-acids composition. IB, SH, BK, JM and AT analysed genes involved in adaptation to extreme environment. NG, DJ, AT, JM, OP and OL wrote the paper. All authors read and approved the final manuscript.

## Supplementary Material

Additional file 1 **Figure S1**. Size distributions of contigs in the global assembly. Click here for file

Additional file 2 **Figure S2**. Distribution of complete protein lengths. Click here for file

Additional file 3 **Figure S3**. GC content and expression level of ribosomal genes. Click here for file

Additional file 4 **Figure S4**. Example of *Alvinella *proteins mapped on a KEGG pathway. Click here for file

Additional file 5 **Figure S5**. Amino acid composition across model taxa. Click here for file
