# Supplementary material for: Insights into metazoan evolution from alvinella pompejana cDNAs
Source: BMC Genomics. 2010 Nov 16;11:634. doi: 10.1186/1471-2164-11-634 (PMC3018142; doi:10.1186/1471-2164-11-634)

Supplemental Figure S1.

Size distribution of the 4,993 contigs and 10,865 singletons resulting from the global assembly of 76,134 clones. Frequencies of singletons and contigs are indicated in blue for the full set of sequences, in red for sequences without CDS, in green for CDS with no detected similarity and in purple for CDS with homologs. We used a logarithmic scale for representation convenience.

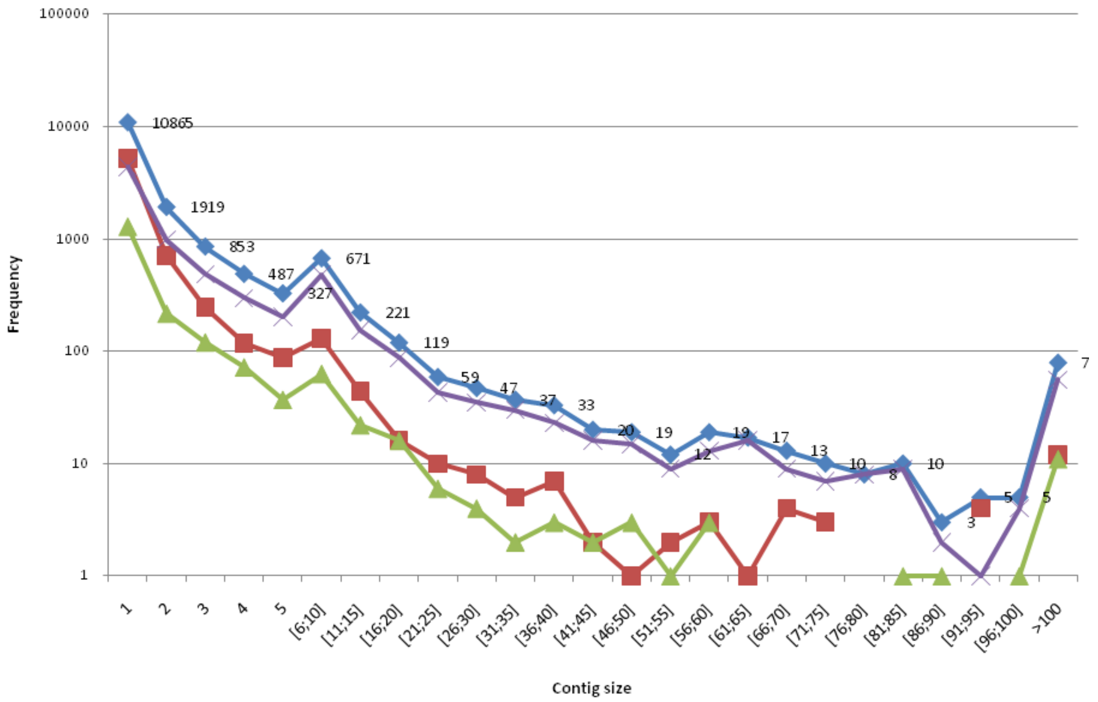

Supplement: Additional file 1 — Figure S1. Size distributions of contigs in the global assembly. [file 1471-2164-11-634-S1.PDF]
