# Supplementary material for: Insights into metazoan evolution from alvinella pompejana cDNAs
Source: BMC Genomics. 2010 Nov 16;11:634. doi: 10.1186/1471-2164-11-634 (PMC3018142; doi:10.1186/1471-2164-11-634)

Supplemental Figure S2.

Distribution of complete protein lengths. Frequencies of the whole set of complete proteins are indicated in blue. Frequencies of complete proteins with and without homologs are indicated in green and red respectively.

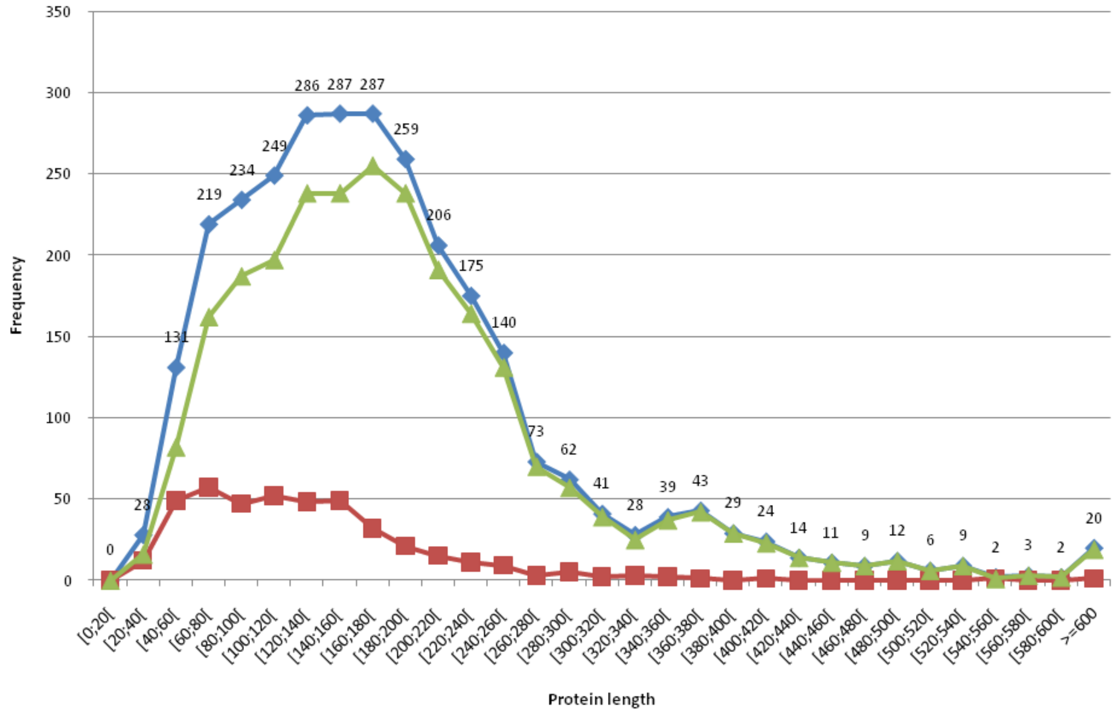

Supplement: Additional file 2 — Figure S2. Distribution of complete protein lengths. [file 1471-2164-11-634-S2.PDF]
