# Supplementary material for: Insights into metazoan evolution from alvinella pompejana cDNAs
Source: BMC Genomics. 2010 Nov 16;11:634. doi: 10.1186/1471-2164-11-634 (PMC3018142; doi:10.1186/1471-2164-11-634)

Supplemental Figure S3

GC content and expression level of ribosomal genes. GC3 (CDS) and GC (UTRs) contents are indicated in black and white respectively.

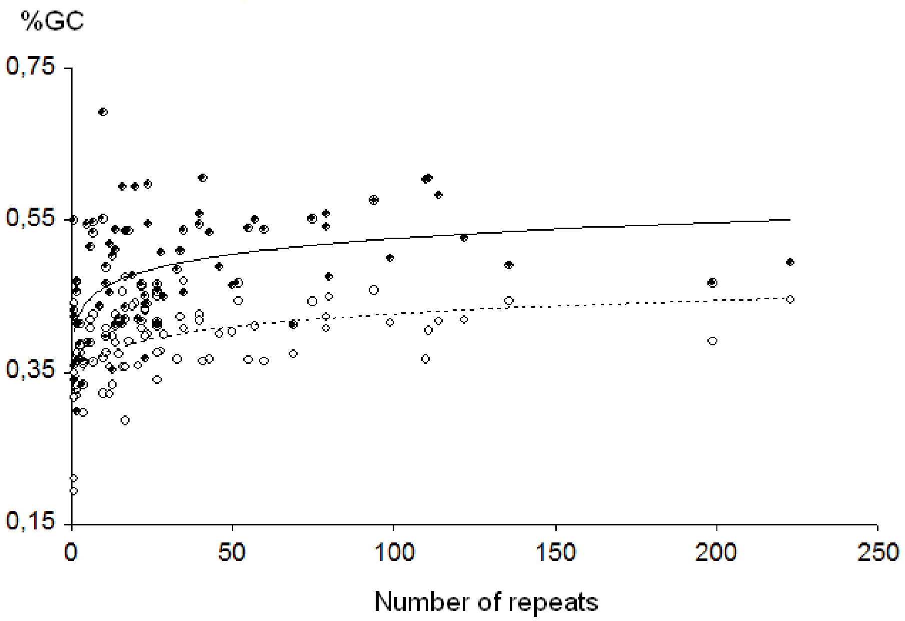

Supplement: Additional file 3 — Figure S3. GC content and expression level of ribosomal genes. [file 1471-2164-11-634-S3.PDF]
