# Supplementary material for: Insights into metazoan evolution from alvinella pompejana cDNAs
Source: BMC Genomics. 2010 Nov 16;11:634. doi: 10.1186/1471-2164-11-634 (PMC3018142; doi:10.1186/1471-2164-11-634)

Supplemental Figure S4.

KEGG map of the steroid biosynthesis pathway (http://www.genome.jp/kegg/pathway.html). Enzyme Classification numbers represented in our *Alvinella* libraries are indicated in orange.

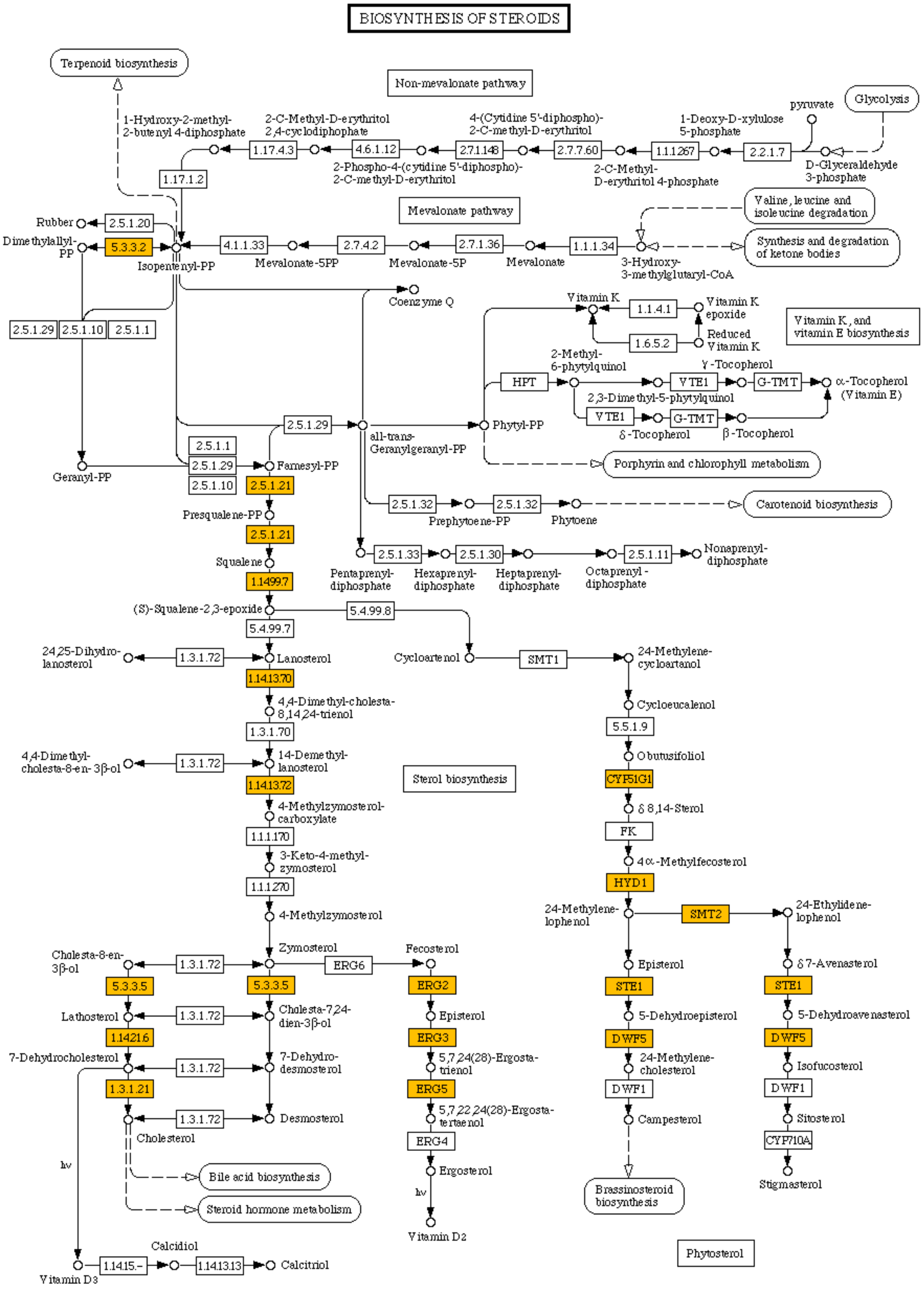

Supplement: Additional file 4 — Figure S4. Example of Alvinella proteins mapped on a KEGG pathway. [file 1471-2164-11-634-S4.PDF]
