# Supplementary material for: Insights into metazoan evolution from alvinella pompejana cDNAs
Source: BMC Genomics. 2010 Nov 16;11:634. doi: 10.1186/1471-2164-11-634 (PMC3018142; doi:10.1186/1471-2164-11-634)

Supplemental Figure S5- Amino acid composition across model taxa

The y-axis indicates the proportion of charged amino acids, the x-axis represents the hydrophobicity index.

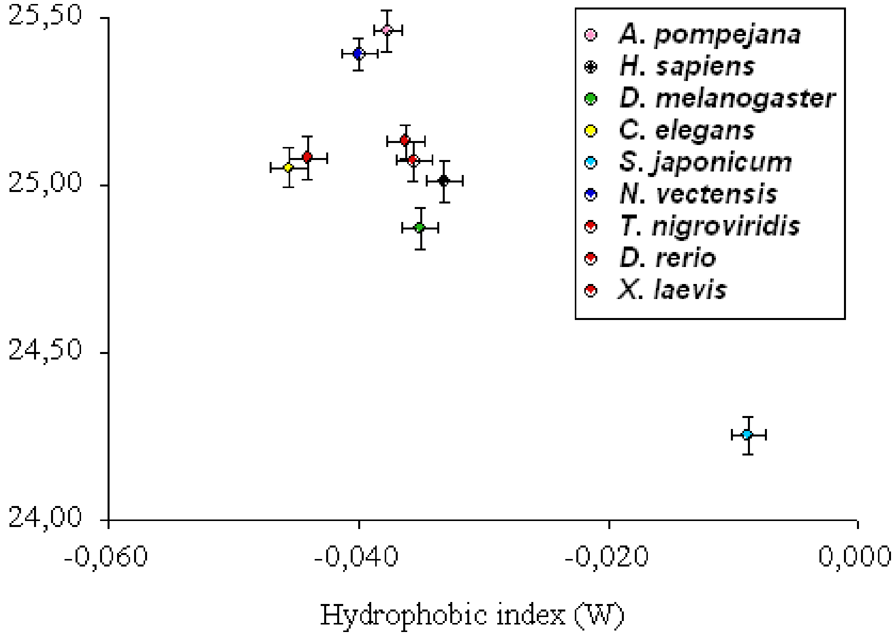

Supplement: Additional file 5 — Figure S5. Amino acid composition across model taxa. [file 1471-2164-11-634-S5.PDF]
